# Low‐level EFCAB1 promoted progress by upregulated DNMT3B and could be as a potential biomarker in lung adenocarcinoma

**DOI:** 10.1002/jcla.24166

**Published:** 2021-12-14

**Authors:** Xiang Yang, Wenjing Shi, Xiaolou Huang, Lijuan Hu, Junjun Wang, Fan Zhang, Yumin Wang, Kate Huang

**Affiliations:** ^1^ Department of Laboratory Medicine The First Affiliated Hospital of Wenzhou Medical University Wenzhou China; ^2^ Department of Pathology The First Affiliated Hospital of Wenzhou Medical University Wenzhou China

**Keywords:** biomarker, EFCAB1, lung adenocarcinomas, methylation

## Abstract

**Background:**

Lung adenocarcinoma (LUAD) incidence is on the rise. We found that EFCAB1 (EF‐Hand Calcium Binding Domain 1) was significantly downregulated in LUAD tissues, but the mechanism of EFCAB1 is unknown.

**Methods:**

One hundred and two LUAD samples and corresponding NT samples were prospectively collected from patients at the First Affiliated Hospital of Wenzhou Medical University, Wenzhou, China, from August 2018 to August 2021.EFCAB1 expression was estimated in LUAD cells and tissues by qPCR. In‐vitro cytology assays were used to detect the role of EFCAB1 in LUAD cells.

**Results:**

EFCAB1 expression level of LUAD was significantly lower than it's adjacent cancer tissues and that of LUAD with big tumor size (>2 cm) was significantly lower than that of small tumor size (≤2 cm) group. It shown that expression levels of EFCAB1 from A549, HCC827, PC9 were lowly expressed. The cell migration, invasion, colony formation, proliferation ability of EFCAB1 OE A549, PC9 were lower than that of EFCAB1 OE A549, PC9 NC group, while the apoptotic cells percentage of the EFCAB1 OE A549, PC9 group were significantly increased. We found that DNMT1 mRNA expression level of PC9 was higher than that of BEAS‐2B, while these of A549, HCC827 decreased. Compared with BEAS‐2B, DNMT3A mRNA expression level of PC9 increased. DNMT3B mRNA expression level of PC9, HCC827 were higher than these of BEAS‐2B.

**Conclusion:**

The EFCAB1 mRNA in LUAD patients and cell lines were downregulated; EFCAB1 overexpression inhibited cell proliferation, migration, invasion, while promoted apoptosis. EFCAB1 was expected to become a biomarker of LUAD.

## Graphical abstract

The expression of EFCAB1 mRNA in LUAD patients and cell lines showed low expression; overexpression of EFCAB1 significantly inhibited cell proliferation, migration, invasion, and apoptosis. DNMT3B is promoting the occurrence of EFCAB1 methylation.

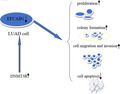

## 1 INTRODUCTION

Despite the continuous improvements in surgical resection, chemotherapy and radiotherapy techniques, lung cancer is still highly prone to recurrence and fatal.^1^ Non‐small cell lung cancer (NSCLC) is the most common in lung cancer, accounting for about 80% of the total number of lung cancers. Because the biological behavior of NSCLC is highly invasive and highly metastatic, about 80% of patients are at an advanced stage at the time of diagnosis, which often leads to treatment failure and death. At present, the overall effect of NSCLC treatment is still very unsatisfactory, and the 5‐year survival rate is only 10%–15%.^2^ Lung adenocarcinoma (LUAD) is a type of NSCLC, which is more likely to occur in women and non‐smokers, and its incidence is on the rise. At present, the pathogenesis of lung cancer is not clear, so further research on LUAD is of great significance. Therefore, looking for more specific molecular targets and biomarkers for early diagnosis of LUAD has become one of the current hot spots in clinical research at home and abroad.

In order to further explore the relevant mRNAs genes in the process of lung cancer, in the previous work, we have used high‐throughput mRNA chip technology to compare the differential mRNA expression profiles between LUAD and its adjacent tissues, screened and initially identified a batch of mRNA molecules associated with LUAD. Integrating multiple screening conditions such as multiple difference, gene locus comparison, and adjacent coding gene information analysis, we preliminarily identified several candidate genes for mRNAs of interest and verified them with real‐time fluorescent quantitative RT‐PCR, and found that the expression of multiple mRNAs and microarrays. The results are consistent, indicating that there are indeed many mRNAs expression disorders in the development of LUAD. Among them, EFCAB1 (EF‐Hand Calcium Binding Domain 1) is the mRNA whose expression in these candidate genes is significantly downregulated. The gene is located at 8q11.21, and the mRNA length is 2286 bp. There is no research report on EFCAB1 and lung cancer at home and abroad. Ohara K et al. analyzed 1007 non‐cancerous tissue (N) samples and corresponding cancer tissue (T) data from lung cancer, stomach cancer, kidney cancer, breast cancer, and liver cancer by single CpG resolution Infinium assay. Principal component analysis shown that *N* samples of each organ shown different DNA methylation profiles, the DNA methylation profiles of N samples of each organ were inherited by the corresponding T samples and the DNA methylation profiles of T samples. It was more like the DNA methylation profiles of N samples and found that EFCAB1 was one of the genes that are highly methylated and extremely low expressed.^3^ Therefore, we speculated that EFCAB1 might play an important role in LUAD due to its low‐level expression through promoter methylation. The specific mechanism is unknown.

In the present study, EFCAB1 expression levels were estimated by quantitative PCR in 60 pairs of LUAD and NT samples and the relationship between EFCAB1 and clinical data from LUAD patients was analyzed. We overexpressed EFCAB1 and observed changes in the oncological behavior of A549 and PC9 cells.

## 2 MATERIALS AND METHODS

### 2.1 Patient samples

One hundred and two LUAD samples and corresponding NT samples were prospectively collected from patients at the First Affiliated Hospital of Wenzhou Medical University, Wenzhou, China, from August 2018 to October 2021. The diagnosis of adenocarcinoma was confirmed by histopathology. Among them, 56 were female and 46 were male, aged 23–81 years. LUAD and matched NT samples were snap frozen in liquid nitrogen immediately after resection based on the TNM clinical staging established by the American Joint Committee on Cancer (AJCC) and the Union for International Cancer Control (UICC) in 2002. Survival data were divided into high and low expression groups according to the expression level of EFCAB1. The study was approved by the ethical review committee of the First Affiliated Hospital of Wenzhou Medical University, and all patients signed written informed consent for this study.

### 2.2 Quantitative PCR

Total RNA was extracted from frozen LUAD tissues using TRIzol reagent (Invitrogen). Total RNA was reverse transcribed to cDNA using RT Reagent Kit (Shanghai Takara) according to the manufacturer's instructions. Gene mRNA expression in LUAD tissues was measured by qPCR using SYBR Premix Ex Taq in an ABI 7000 instrument. The qPCR was used to measure gene mRNA expression in LUAD tissues. The detailed primer sequences for qPCR are shown in Table 1. Total RNA (2 mg) was transcribed into cDNA. The total volume of the PCR reaction was 20 μl, including 10 μl SYBR Premix (2×), 2 μl cDNA template, 1 μl PCR forward primer (10 mM), 1 μl PCR reverse primer (10 mM), and 6 μl double‐stranded distilled water. The qPCR reaction consists of an initial denaturation step at 95°C for 10 min; 40 cycles of 95°C for 5 s and 60°C for 30 s; and a final extension at 72°C for 5 min. All experiments were performed in triplicate and all samples were normalized for β‐actin. The median of each triplicate was used to calculate the relative mRNA concentration (△Ct = Ct median lncRNA‐Ct median GAPDH), and 2^−△△Ct^ in expression were calculated.^4^

**TABLE 1:** Detailed primer sequences for real‐time RT‐PCR

Gene	Primer sequences PCR product (bp)	
	Forward	Reverse
EFCAB1	GGAGTAGAGAGGCAAGGTCTG	TATCAAAACCTCGGAATACTCTGT 167
β‐actin	CCTGGCACCCAGCACAAT	GCTGATCCACATCTGCTGGAA 158
DNMT1	GCGGCTCAAAGATTTGGAAAGA	CCAGGTAGCCCTCCTCGGAT 161
DNMT3B	GTCGTGCAGGCAGTAGGAAAT	GAAGCCATTTGTTCTCGGCT 178
DNMT3A	CGCGATTTCTCGAGTCCAAC	TTGGCTATCCTGCCATGCTC 169

### 2.3 Biochemical analysis

Collected 5 ml of fasting blood from the patient, centrifuged at 3140 g*5 min, aspirated the supernatant into an EP tube and stored in the refrigerator at −80°C. The electrochemiluminescence method was used to detect the patient's serum carcino‐embryonic antigen (CEA), neuron‐specific enolase (NSE), Cytokeratin‐19‐fragment (CYFRA21‐1) levels. The reagents, quality control materials and calibration solutions were all made by Roche.

### 2.4 Cell culture

Normal human bronchial epithelial cells BEAS‐2B and 3 individual LUAD cell lines (A549, HCC827, PC9) were purchased from the cell bank of Chinese Academy of Sciences and cultured in complete medium (containing 10% fetal serum and 90% RPMI1640) at 37°C, 5% CO2, with complete medium changed at least once every 2 days.

### 2.5 Transfection

Plasmid pcDNA‐EFCAB1 and control vector pcDNA‐3.1 were purchased from Suzhou Emax Biotechnology Company. Lipofectamine 3000 (Invitrogen) was used for cell transfection.

### 2.6 Cell viability assay

Cell viability was assessed by Cell Counting Kit‐8 (CCK‐8, Corning Corporation) according to the manufacturer's protocol. Briefly, 3000 cells were resuspended and inoculated into 96‐well plates supplemented with 10% FBS and cultured for 1 week. The next day, EFCAB1 overexpression cells were incubated with CCK‐8 for 1 h and absorbance was measured at 450 nm on days 1, 3, 5, and 7 using a multifunctional enzyme marker (Tecan). The experiment was performed in quadruplicate cells.

### 2.7 Cell migration and invasion assays

Migration and invasion assays were performed in 24‐well plates with 8.0‐μm well inserts (Millipore). For migration assays, 2 × 10^4^ cells were inoculated into the upper compartment of the transwell insert. Invasion assays were performed with a matrix gel‐coated filter (Sigma Corporation). Cells were allowed to incubate for 24 and 48 h, respectively. Migration and invasion were fixed with methanol and stained with 0.1% (w/v) crystalline violet, followed by bleaching with 33% acetic acid and measuring the 570 nm absorbance values on an enzyme marker. Each experiment was performed in triplicate.

### 2.8 Colony formation assay

Six hundred cells/well were inoculated in six‐well plates, cultured for 14 days, fixed, stained, and observed for cell colony formation. The number of cell clones was compared to determine the ability of cell clone formation.

### 2.9 Flow cytometry to detect apoptosis

Apoptosis detection kit (KeyGEN) was used for apoptosis detection. After washing the cells twice with PBS, the cells were resuspended with 500 µl Binding Buffer, 5 µl Annexin V‐APC and 5ul 7‐AAD dye solution were added and the reaction was performed for 5–15 min after protection from light in a flow cytometer (Beckman).

### 2.10 Statistical methods

Comparisons between the two groups were performed by Student's *t* test. Survival analysis was performed using the chi‐square test. *p *< 0.05 was statistically significant.

## 3 RESULTS

### 3.1 The expression level of EFCAB1 in lung cancer and adjacent tissues and analysis of its relationship with clinical data

**FIGURE 1:**
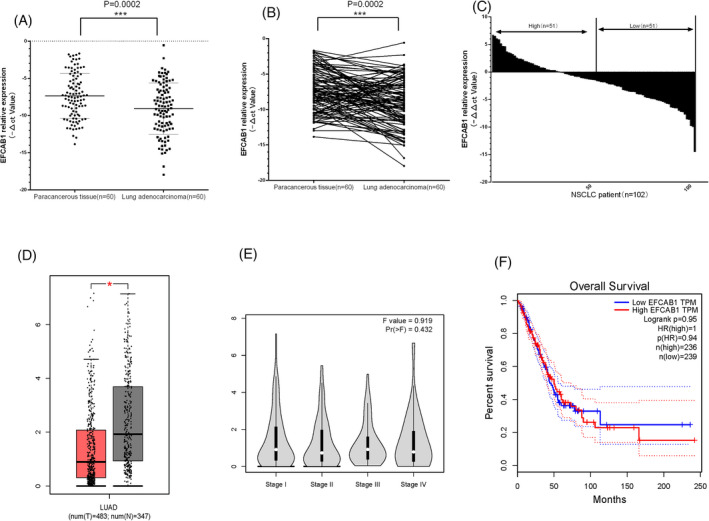
Expression level of EFCAB1 in lung cancer and adjacent tissues. (A) EFCAB1 expression level of LUAD was significantly lower than its adjacent cancer tissues (*p *= 0.0002) in scatter graph. (B) EFCAB1 expression level of LUAD was significantly lower than its adjacent cancer tissues (*p *= 0.0002) in line graph. (C) EFCAB1 expression level of LUAD was significantly lower than its adjacent cancer tissues in data bar graph. (D)It showed that the level of EFCAB1 mRNA in LUAD was significantly lower than that of adjacent tissues from GEPIA data (http://gepia.cancer‐pku.cn/index.html). (E)It showed that the level of EFCAB1 mRNA in LUAD was not related to clinical stage. (F)Further analysis showed that the prognosis of LUAD was not significantly different from the high or low level of EFCAB1 mRNA expression

According to Figure 1A–C, EFCAB1 expression level of LUAD was significantly lower than its adjacent cancer tissues (Figure 1A–C). We showed that the EFCAB1 level of LUAD with big tumor size (>2 cm) was significantly lower than that of small tumor size (≤2 cm) group. EFCAB1 expression levels among different clinical parameters [Age, Gender, TNM stage, carcino‐embryonic antigen (CEA), neuron‐specific enolase (NSE), and Cytokeratin‐19‐fragment (CYFRA21‐1)] were not different (*p *= 0.634 *p *= 0.426, *p *= 0.262, *p *= 0.336, *p *= 0.501, *p *= 0.065, *p *= 0.401) in Table 1. We analyzed the expression of EFCAB1 in lung adenocarcinoma from GEPIA data (http://gepia.cancer‐pku.cn/index.html), and the results showed that the level of EFCAB1 mRNA in LUAD was significantly lower than that of adjacent tissues (Figure 1D), but it was not related to clinical stage (Figure 1E), which is consistent with our results. Further analysis showed that the prognosis of LUAD was not significantly different from the high or low level of EFCAB1 mRNA expression (Figure 1F).

### 3.2 The expression level of EFCAB1 from three LUAD cells and constructed the A549 EFCAB1 OE group and PC9 EFCAB1 OE group

**FIGURE 2:**
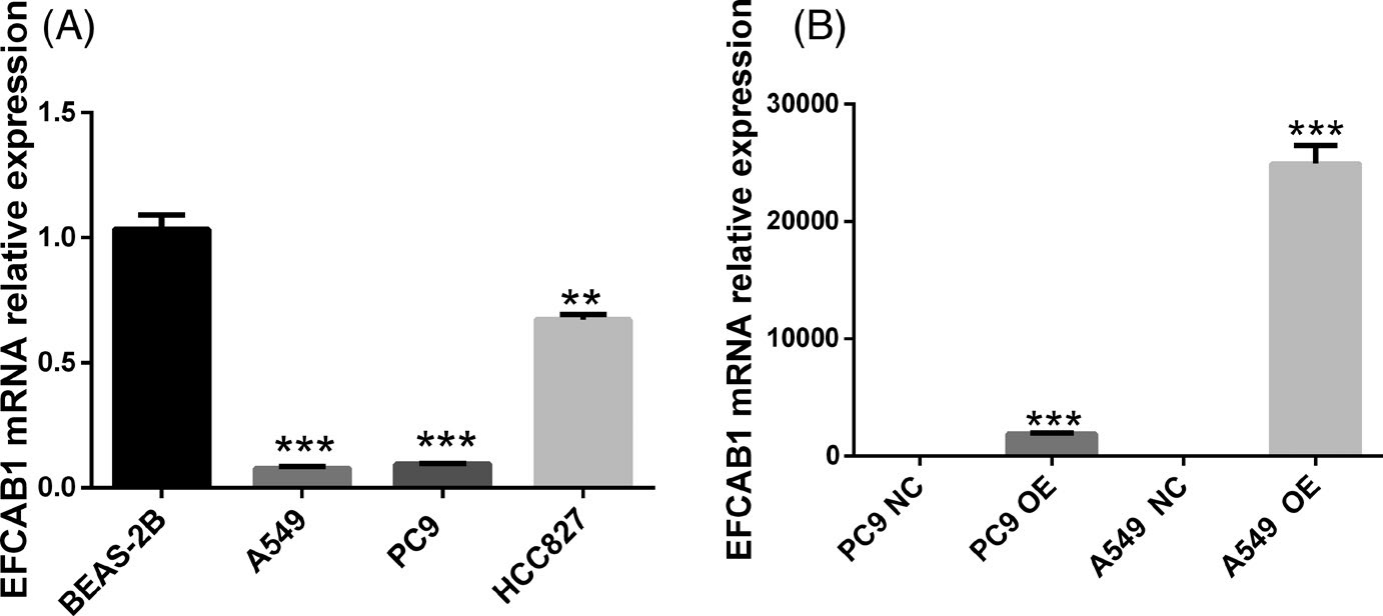
Expression level of EFCAB1 from three LUAD cells and constructed the A549 EFCAB1 OE group and PC9 EFCAB1 OE group. (A) It shown that the expression levels of EFCAB1 from A549 (*p *< 0.001), HCC827 (*p *< 0.01), PC9 (*p *< 0.001) cells were lowly expressed than BEAS‐2B cell line. (B) QPCR results after transfection showed that the expression levels of A549 EFCAB1 OE group (*p *< 0.001) and PC9 EFCAB1 OE group (*p *< 0.001) were significantly higher than those of A549 EFCAB1 OE NC group and PC9 EFCAB1 OE NC group

Compared to normal human bronchial epithelial BEAS‐2B cell line, we detected the expression levels of EFCAB1 from three LUAD cell lines (including A549, HCC827, PC9) by qPCR. It is shown that the expression levels of EFCAB1 from A549, HCC827, PC9 cells were lowly expressed than BEAS‐2B cell line, and seen in Figure 2A. Therefore, we choose PC9 and A549 cells to be co‐transfected with the overexpression plasmid; the qPCR results showed that the expression levels of A549 EFCAB1 OE group and PC9 EFCAB1 OE group after transfection were significantly higher than those of A549 EFCAB1 OE NC group and PC9 EFCAB1 OE NC group and seen in Figure 2B. It shown that we successfully constructed the A549 EFCAB1 OE group and PC9 EFCAB1 OE group (Table 2).

**TABLE 2:** The relationship between the expression level of EFCAB1 and the clinical characteristics of lung adenocarcinoma

Parameter	*N* (102)	Relative EFCAB1 expression		
		Low (51)	High (51)	*p* value
Age/years				0.634
≤60	46	21	25	
>60	56	30	26	
Gender				0.426
Male	46	25	21	
Female	56	26	30	
Tumor size				0.000
≤2 cm	59	18	41	
>2 cm	43	33	10	
Lymph node metastasis				0.262
Negative	75	40	35	
Positive	27	11	16	
TNM stage				0.336
I stage	80	42	38	
Ⅱ–Ⅳ stage	22	9	13	
CEA (ng/ml)				0.501
≤5	75	36	39	
>5	27	15	12	
CYFRA21‐1 (ng/ml)				0.065
≤3.3	73	38	35	
>3.3	29	13	16	
NSE (ng/ml)				0.401
≤15	68	32	36	
>15	34	19	15	

### 3.3 EFCAB1 inhibited cell migration and invasion in LUAD

**FIGURE 3:**
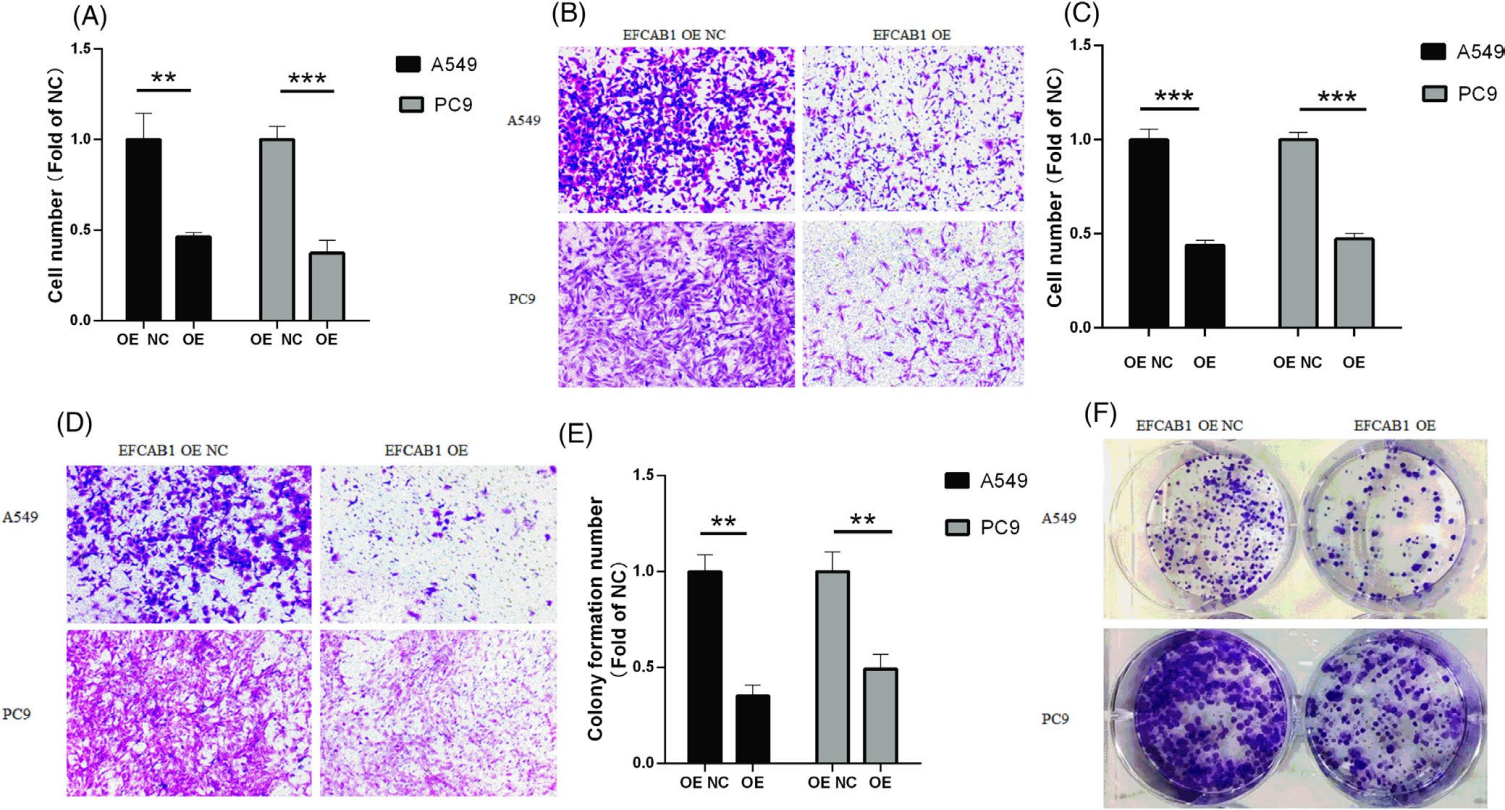
EFCAB1 inhibited cell migration, invasion and cell clone information in LUAD. (A)The cell migration shown that cell numbers of EFCAB1 OE A549, PC9 were lower than that of EFCAB1 OE A549, PC9 NC group (*p *< 0.01, *p *< 0.001). (B)The cell migration graphs of EFCAB1 OE A549, PC9, EFCAB1 OE A549, PC9 NC group. (C) the cell numbers of EFCAB1 OE A549, PC9 were lower than that of EFCAB1 OE A549, PC9 NC group (*p *< 0.001, *p *< 0.001), it hinted that EFCAB1 inhibited cell invasion ability of LUAD. (D)The cell invasion graphs of EFCAB1 OE A549, PC9, EFCAB1 OE A549, PC9 NC group. (E) Compared to EFCAB1 OE A549, PC9 NC group, colony formation number of EFCAB1 OE A549, PC9 appeared as a significant decline. (F) The cell colony formation graphs of EFCAB1 OE A549, PC9, EFCAB1 OE A549, PC9 NC group

The cell migration shown that cell numbers of EFCAB1 OE A549, PC9 were lower than that of EFCAB1 OE A549, PC9 NC group (*p *< 0.01, *p *< 0.001), see Figure 3A,B. So EFCAB1 inhibited cell migration of LUAD. According to Figure 3C,D, the cell numbers of EFCAB1 OE A549, PC9 were lower than that of EFCAB1 OE A549, PC9 NC group (*p *< 0.001, *p *< 0.001), it hinted that EFCAB1 inhibited cell invasion ability of LUAD.

### 3.4 EFCAB1‐reduced ability of cell clone information and proliferation in LUAD

**FIGURE 4:**
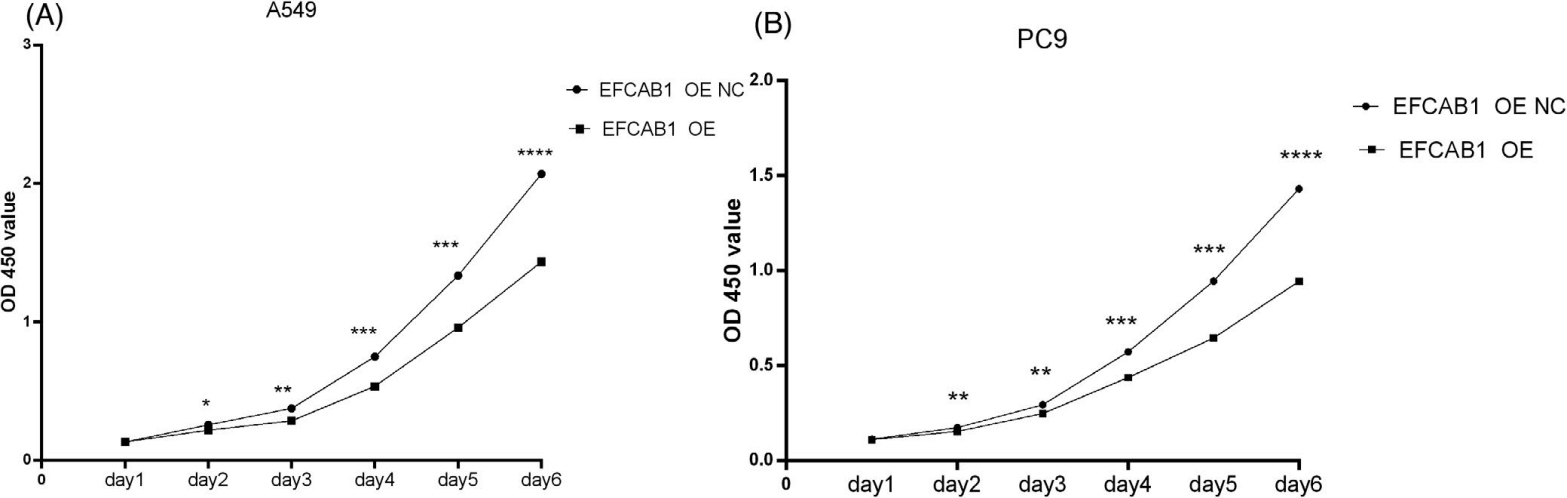
EFCAB1‐reduced proliferation ability in LUAD. (A)The OD450nm of different A549 groups gradually increased with the change of time. Compared with appropriate days of EFCAB1 OE A549 NC group, the OD450 nm of 1 day in EFCAB1 OE A549 group were no statistically significant difference (*p* > 0.05), while that of the 2 days (*p *< 0.05), the 3 days (*p *< 0.01), the 4 days (*p *< 0.001), the 5 days (*p *< 0.001) and the 6 days (*p *< 0.0001) significantly reduced. (B) The OD450nm of different PC9 groups gradually increased with the change of time. Compared with appropriate days of EFCAB1 OE PC9 NC group, the OD450 nm of 1 day in EFCAB1 OE PC9 group were no statistically significant difference (*p* > 0.05), while that of the 2 days (*p *< 0.01), the 3 days (*p *< 0.01), the 4 days (*p *< 0.0001), the 5 days (*p *< 0.001) and the 6 days (*p *< 0.0001) significantly reduced

In the Figure 3E,F, compared to EFCAB1 OE A549, PC9 NC group, colony formation number of EFCAB1 OE A549, PC9 appeared as a significant decline, after EFCAB1 was overexpressed and demonstrated that EFCAB1‐reduced ability of cell clone information of LUAD. In the Figure 4A,B, the OD450nm of different A549 groups gradually increased with the change of time. Compared with appropriate days of EFCAB1 OE A549, PC9 NC group, the OD450 nm of 1 day in EFCAB1 OE A549, PC9 group were no statistically significant difference, while that of the 2, 3, 4, 5, and 6 days, it significantly reduced, it indicated that cell proliferation ability of A549, PC9 was significantly reduced after EFCAB1 overexpressed.

### 3.5 EFCAB1 promoted the ability of cell apoptosis in LUAD

**FIGURE 5:**
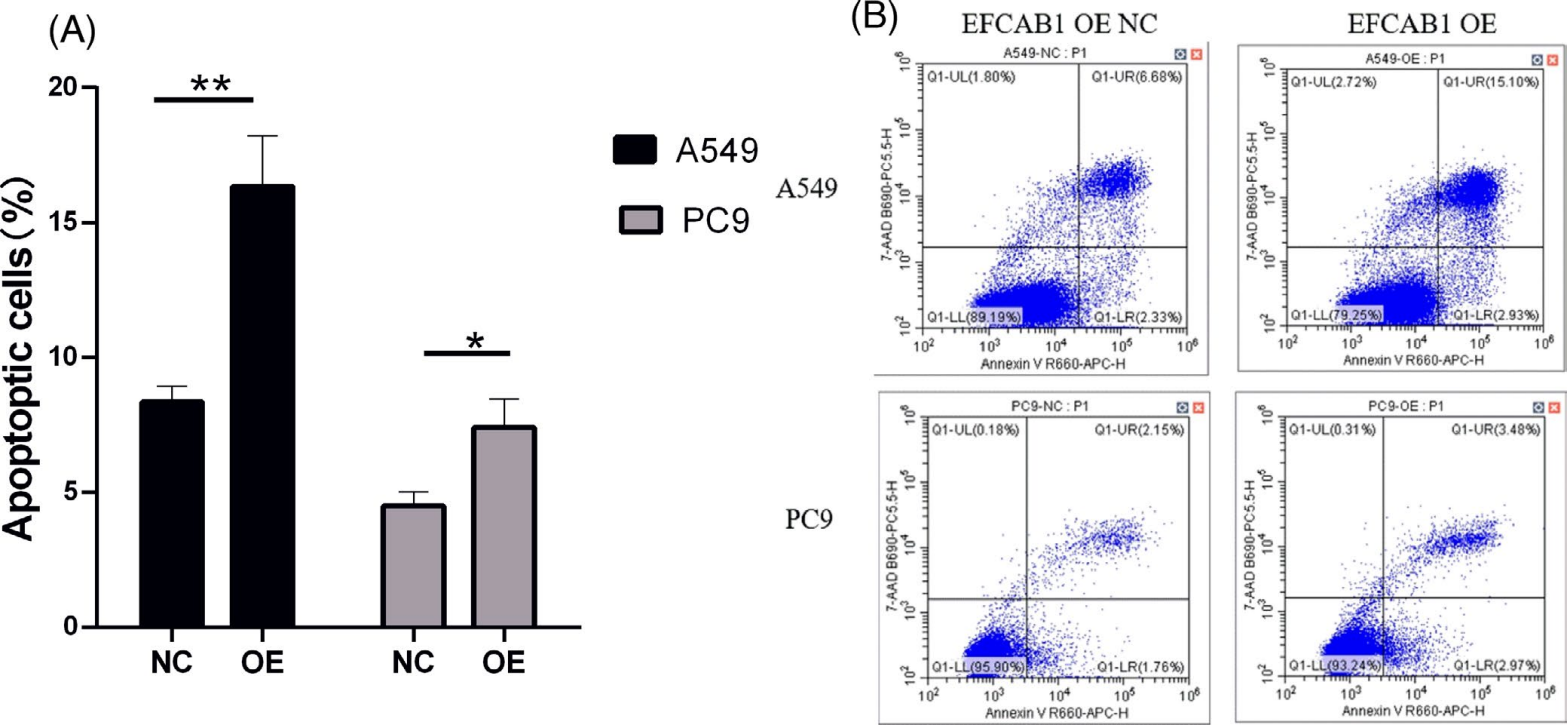
EFCAB1 expression level was associated with the ability of cell apoptosis in LUAD. (A)Compared with the EFCAB1 OE A549 (8.34% ± 0.34%) NC group, the apoptotic cells percentage of the EFCAB1 OE A549 (16.33% ± 1.09%) group was significantly increased. (B) Compared with the EFCAB1 OE PC9 (4.50% ± 0.30%) NC group, the apoptotic cells percentage of the EFCAB1 OE, PC9 (7.41% ± 0.60%) group was significantly increased

Compared with the EFCAB1 OE A549 (8.34% ± 0.34%), PC9 (4.50% ± 0.30%) NC group, the apoptotic cells percentage of the EFCAB1 OE A549 (16.33% ± 1.09%, *p *< 0.01), PC9 (7.41% ± 0.60%, *p *< 0.05) group were significantly increased. (Figure 5A,B).

### 3.6 DNMT3B might be involved in the methylation of LUAD with low expression of EFCAB1

**FIGURE 6:**
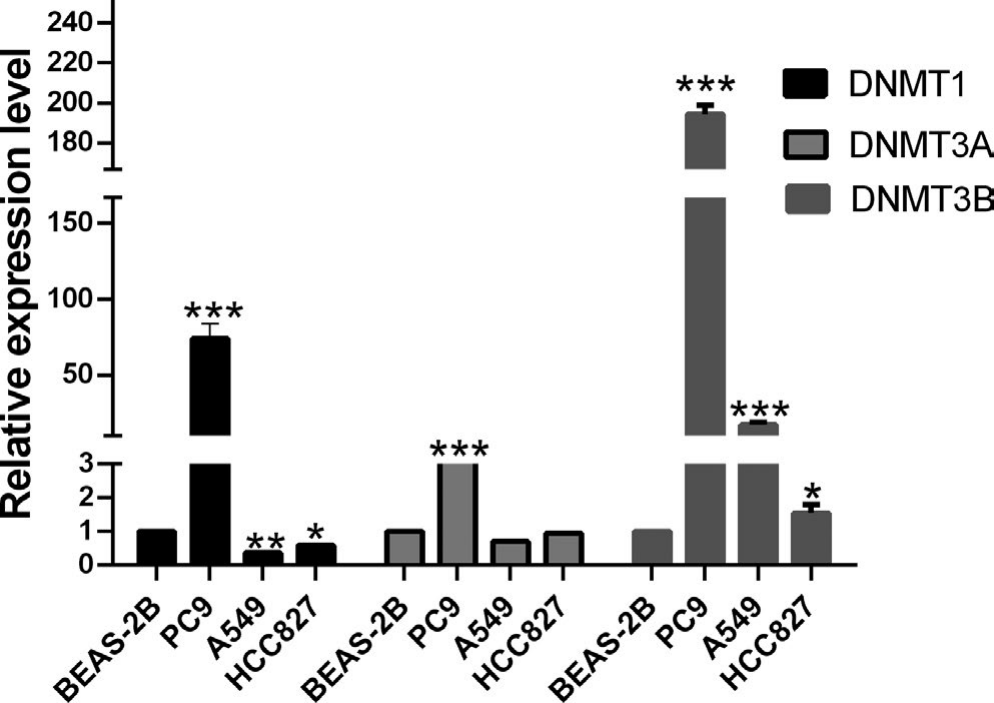
DNMT3B might be involved in the methylation of LUAD with low expression of EFCAB1. We found that DNMT1 mRNA expression level of PC9 (*p *< 0.001) was higher than that of BEAS‐2B, while these of A549 (*p *< 0.01), HCC827 (*p *< 0.05) were lower than these of BEAS‐2B. Compared with BEAS‐2B, DNMT3A mRNA expression level of PC9 (*p *< 0.001) increased, while these of A549 (*p *> 0.05), HCC827 (*p *> 0.05) cell lines were no statistical difference. DNMT3B mRNA expression level of PC9 (*p *< 0.001), HCC827 (*p *< 0.001, *p *< 0.001, *p *< 0.05) cell lines were higher than these of BEAS‐2B, while A549 was no statistical difference

According to the literature,^3^ we speculated that EFCAB1 was hypermethylated and the expression level was low. The methylation pattern of DNA in the genome is realized by DNA methyltransferase. DNMT1, DNMT3A, and DNMT3B are currently relatively common DNA methyltransferases.^5^, ^6^ In the Figure 6, we found that DNMT1 mRNA expression level of PC9 was higher than that of BEAS‐2B, while these of A549, HCC827 were lower than these of BEAS‐2B. Compared with BEAS‐2B, DNMT3A mRNA expression level of PC9 increased, while these of A549, HCC827 cell lines were no statistical difference. DNMT3B mRNA expression level of PC9, HCC827 cell lines were higher than these of BEAS‐2B, while A549 was no statistical difference. Combined with the results of Figure 2A, we speculated that DNMT3B might play an important role of promoting the occurrence of EFCAB1 methylation.

## 4 DISCUSSION

Calcium‐binding protein EFCAB1 is a member of the calcium‐binding EF‐hand structural domain family of proteins. EFCAB1 was found to be present in sea urchin spermatozoa and binds to calcium ions to alter axoneme conformation and thereby regulate sperm motility.^7^ Zhu et al. found that upregulated expression of EFCAB1 in breast cancer tissues was also observed in GSE33447. Higher expression of miR‐1908 indicated a poorer prognosis, whereas higher expression of EFCAB1 was associated with a better prognosis of breast cancer. Based on these findings a potential miR‐1908‐3p‐mRNA regulatory network, miR‐1908‐3P‐EFCAB1, could be established to contribute to the development and progression of breast cancer.^8^

In this study, we found that the expression level of EFCAB1 in LUAD was significantly lower than that of its adjacent cancer tissues, and the expression level of LUAD with large tumors (>2 cm) was significantly lower than that with small tumors (≤2 cm). Compared with BEAS‐2B cell lines, A549, HCC827, and PC9 have lower expression levels of EFCAB1. This indicated that EFCAB1 was under‐expressed in LUAD tissues and cell lines, and we have confirmed that it was under‐expressed through TCGA data. EFCAB1 mRNA expression level was relative to progression of LUAD. We further confirmed through cytological experiments that the cell migration, invasion, colony formation and proliferation ability of A549 and PC9 decreased obviously while apoptotic ability increased markedly after EFCAB1 overexpression.

It was more like the DNA methylation profiles of N samples and found that EFCAB1 was one of the genes that are highly methylated and extremely low expressed.^3^ There are two types of DNMTs that methylate DNA: DNMT1, a persistent DNA methyltransferase that acts on DNA duplexes with only one methylated strand to completely methylate them and may be involved in the methylation of new synthetic strands in DNA replication duplexes, DNM T1 may act directly with HDAC (histone deacetyl transferase) to block transcription; DNMT3A and DNMT3B, slave methyltransferases, which methylate CpG, hemi methylate it, and then fully methylate it. DNMT3B plays an important role in oncogene methylation. RAD9 was epigenetically regulated by DNMT1 and DNMT3B, and subsequent RAD9 overproduction promoted prostate tumorigenesis by targeting hypermethylation.^9^ In cells overexpressing miR‐29b, the methylation signature of the promoter of the cell cycle inhibitor gene CDKN2B was reduced. MiR‐29b inhibits the progression of cholangiocarcinoma by releasing the inhibition of CDKN2B expression mediated by DNMT3B.^10^ FOXC1 induced CTH promoter DNA hypermethylation through upregulation of DNMT3B to promote proliferation and metastasis in primary hepatocellular carcinoma.^11^ We found that the expression level of DNMT1 mRNA of PC9, A549, and HCC827 were higher than that of BEAS‐2B, while the expression level of A549 and HCC827 was lower than the expression levels of A549 and HCC827 were lower than those of BEAS‐2B. Compared with BEAS‐2B, PC9 DNMT3A mRNA expression level Increase, while the expression of A549 and HCC827 was not statistically different. The mRNA expression level of DNMT3B was higher than the expression level of BEAS‐2B. So we speculated that DNMT3B might play an important role of promoting the occurrence of EFCAB1 methylation.

In summary, the expression of EFCAB1 mRNA in LUAD patients and cell lines showed low expression; overexpression of EFCAB1 significantly inhibited cell proliferation, migration, invasion, and apoptosis. EFCAB1 is expected to become a biomarker of LUAD.

## CONFLICT OF INTEREST

The authors declare that they have no competing interests.

## Data Availability

Requests for data, resources, and reagents should be directed to the corresponding author Kate Huang (huangkate@wmu.edu.cn).
